# Association between Selenium Status and Chronic Kidney Disease in Middle-Aged and Older Chinese Based on CHNS Data

**DOI:** 10.3390/nu14132695

**Published:** 2022-06-28

**Authors:** Changxiao Xie, Mao Zeng, Zumin Shi, Shengping Li, Ke Jiang, Yong Zhao

**Affiliations:** 1 School of Public Health, Chongqing Medical University, Chongqing 400016, China; 2019110926@stu.cqmu.edu.cn (C.X.); 2019110981@stu.cqmu.edu.cn (S.L.); 2021110631@stu.cqmu.edu.cn (K.J.); 2 Research Center for Medicine and Social Development, Chongqing Medical University, Chongqing 400016, China; 3 Research Center for Public Health Security, Chongqing Medical University, Chongqing 400016, China; 4 Center for Disease Control and Prevention in Shuangliu District of Chengdu, Chengdu 610000, China; zengmao@stu.cqmu.edu.cn; 5 Human Nutrition Department, College of Health Sciences, QU Health, Qatar University, Doha 2713, Qatar; zumin@qu.edu.qa; 6 Chongqing Key Laboratory of Child Nutrition and Health, Children’s Hospital of Chongqing Medical University, Chongqing 400014, China

**Keywords:** selenium, chronic kidney disease (CKD), CHNS, ROC

## Abstract

Background: The association between selenium and chronic kidney disease (CKD) remains controversial. Population studies with large samples facilitate the reliability of conclusions. Objective: In this study, we aimed to describe the prevalence of a CKD association with selenium intake in middle-aged and older Chinese. Methods: Data for this study were obtained from the China Health and Nutrition Survey (CHNS). A total of 5381 participants (aged ≥ 45) with biochemical test data were included in the study. Logistic regression models were used to examine the association between diet selenium intake (quartile) and the prevalence of CKD. Results: A total of 942 (17.01%) participants had CKD. The prevalence of CKD was 23.33%, 20.32%, 14.98%, and 9.25% among participants with average selenium intakes of 21.5 ± 4.82, 33.1 ± 2.79, 43.8 ± 3.70, and 67.0 ± 13.97 µg/day, respectively. In the fully adjusted model (Model 3), across the quartiles of selenium intake, the ORs for the prevalence of CKD were 1.00, 1.09 (95% CI 0.69–1.73), 0.82 (95% CI 0.49–1.38), and 0.43 (95% CI 0.22–0.85). The protein intake had a certain diagnostic significance for the selenium intake. Conclusions: An adequate selenium intake may have a positive effect on CKD. The influence of individual weight and location on the effect of selenium on CKD needs to be further explored.

## 1. Introduction

Chronic kidney disease (CKD) is a chronic kidney structure and function disorder caused by a variety of reasons, of which oxidative stress plays the most important role in its pathogenesis [1]. Globally, in 2017, the prevalence of CKD was 9.1% [2] and 697.5 million cases of CKD were reported. Almost a third of patients with CKD lived in two countries: China (132.3 million cases) and India (115.1 million cases) [2,3]. CKD and its effect on cardiovascular disease resulted in 2.6 million deaths and 35.8 million disability-adjusted life years (DALYs) [2]. It has become a global public health problem with a high disease burden. Diabetes, hypertension, obesity, hyperlipidemia, advanced age, and smoking have been identified as traditional risk factors for CKD [4]. Trace elements such as zinc, manganese, iron, and selenium have also been reported to be involved in the progression of CKD [5,6,7].

In human beings, the nutritional functions of selenium are achieved by 25 selenoproteins that have selenocysteine at their active center [8,9]. Among them, selenium is best known in the form of antioxidant enzymes such as GPx3, the strongest antioxidant enzyme in the body [8,9,10]. The middle-aged and elderly are the populations with a high incidence of various chronic diseases [11]. Studies have shown that a lack of selenium intake was associated with cognitive impairment [12], hypertension [13], and Alzheimer’s disease [14,15]. In addition, considering the physiological characteristics of middle-aged and elderly people with a reduced appetite [16] and digestive capacity [17], they are at a higher risk of an insufficient selenium intake. Middle-aged and elderly people are also susceptible to CKD. The United States Renal Data System report released in 2021 showed that 96.30% of registered ESRD patients in the United States were middle-aged and elderly patients (≥45 years of age) [18].

The kidney, where selenium is most distributed [19], absorbs selenium in plasma through renal tubular epithelial cells to synthesize GPx3 and maintain selenium homeostasis [20,21]. Several studies have shown that selenium levels are associated with the severity of kidney disease [22]. A selenium deficiency causes renal injury through increased oxidative stress and an impaired mitochondrial function [23].

Most of the existing studies on the association between selenium status and CKD focus on the clinical treatment or the molecular level [6,23,24,25] and have controversial results [5,6,24,25]. Selenium is found in different forms in various foods, greatly affecting absorption [26]. Considering that the dietary intake is the main source of selenium for human beings, population research on the association between selenium intake under natural conditions and CKD is lacking. Using data from the China Health and Nutrition Survey (CHNS), we aimed to assess the association between selenium intake and the prevalence of CKD in a Chinese adult population.

## 2. Methods

### 2.1. Study Population

**Figure 1:**
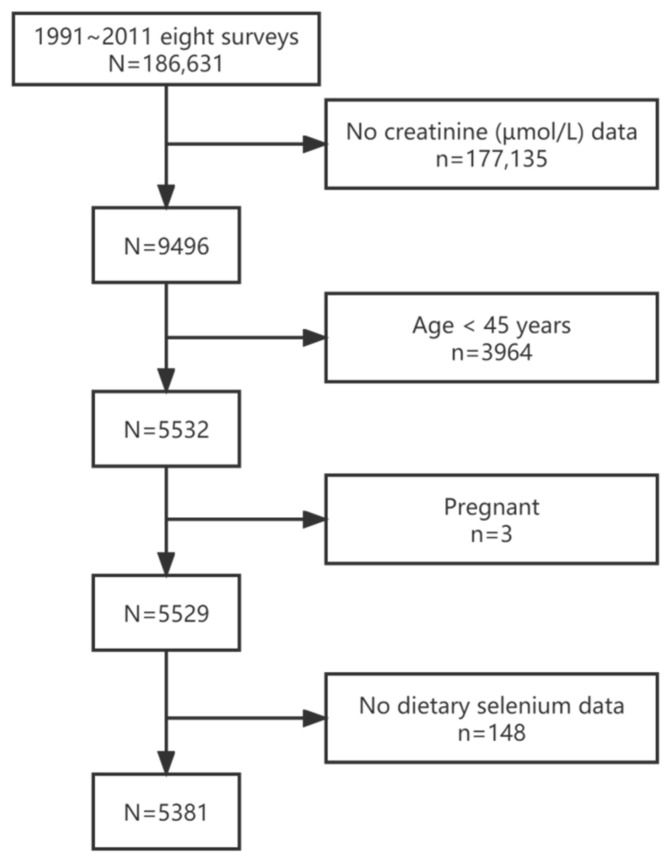
Participant flow chart.

The population was derived from the CHNS, a cohort study established between a large number of scholars from the Chinese Center for Disease Control and Prevention and the University of North Carolina at Chapel Hill [27]. Ten wave surveys were conducted from 1989 to 2015 (1989, 1991, 1993, 1997, 2000, 2004, 2006, 2009, 2011, and 2015). A multistage random cluster process was used to draw the sample surveyed in nine provinces. We used a wide-ranging set of socioeconomic factors (e.g., income, employment, and education), demographic measures (e.g., gender and age), and biomarker data (e.g., creatinine, blood pressure, blood glucose, and total cholesterol) collected in 2009. We selected 2009 with creatinine (µmol/L) data (*n* = 9496) and excluded 3964 participants (<45 years old), 3 pregnant women, and 148 with missing data of selenium intake. Finally, 5381 participants were included (Figure 1).

### 2.2. Outcome Variable: CKD

CKD was defined as an estimated glomerular filtration rate (eGFR) < 60 mL/min per 1.73 m^2^. We used the Chronic Kidney Disease–Epidemiology Collaboration (CKD-EPI) formula [28] to estimate the eGFR. The creatinine level was determined using a colorimetric assay and a Roche Modular P800 instrument [3].

### 2.3. Exposure Variables: Selenium Intake

During the 3 day diet survey, trained investigators weighed and recorded the food and condiments from household stocks, markets, gardens, and leftover food waste. The data were collected using a 3 day/24 h dietary survey. Trained investigators weighed and recorded in detail how much food (including condiments) was stored, how much food was purchased (including home-grown), and how much food was left over at the start, middle, and end of the survey. From this, we obtained the food consumption of family members and calculated the intake information of selenium as well as the total food capacity, carbohydrate, fat, and protein intake according to the Chinese Food Composition Table [29]. On the basis of the description of the same cohort, about 93% of the participants were in a stable state of selenium intake [13], so we only used the dietary intake data from 2009. The selenium intake was recoded into quartiles in the analysis.

### 2.4. Covariates

A structured questionnaire was used to obtain the age (<60 years and ≥60 years); gender (male and female); income as per capita household income (recoded to tertiles as low, medium, and high); urbanization index (recoded to tertiles as low, medium, and high); education (low: illiterate/primary school, medium: junior middle school, high: high middle school or higher and unknown); smoking status (non-smokers, ex-smokers, and current smokers); and alcohol drinking status (yes and no). Physical activity levels were estimated using self-reported activities (including occupation, family, transportation, and recreation) and time spent (metabolic equivalent of task (MET), hours/week) [30].

Physical measurements and a biological sample collection questionnaire were used to obtain the height, weight, systolic blood pressure, diastolic blood pressure, blood glucose level, triglycerides (TGs), cholesterol (TC), low-density lipoprotein (LDL), high-density lipoprotein (HDL), creatinine, and other data. All anthropometric measurements were obtained according to China’s Regulations on Hygienic Standards for Anthropometric Methods (WS/T 424-2013) [31]. We defined the BMI status according to the China cut-off (low: BMI < 18.5, normal: 18.5 ≤ BMI < 24, overweight: 24 ≤ BMI < 28, and obesity: BMI ≥ 28 [32]). Hypertension was defined as a mean of three measurements with systolic blood pressure ≥ 140 mmHg and/or diastolic blood pressure ≥ 90 mmHg without taking hypertension medications. Participants who had been diagnosed with hypertension or who were taking anti-hypertensive drugs were also counted [13]. Participants were labeled as having diabetes when their fasting venous plasma glucose ≥ 7.0 mmol/L or if they had been diagnosed with diabetes [33]. The National Cholesterol Education Program Adult Treatment Panel III (NCEP-ATPIII) was used to define hyperlipidemia [34]. In addition, all dietary intake information calculated from the Chinese food composition list was included as covariates.

### 2.5. Statistical Analyses

Stata16.0 (Stata Corporation, College Station, TX, USA) was used for all statistical analyses. We defined the data beyond 1–99% of the data of the energy intake and nutrient intake as outliers and treated them as missing. In the descriptive analysis, the continuous variables were described by the mean (standard deviation) and the categorical variables were described by the number (percentage). Binary logistic regression models were used to analyze the association between selenium and CKD. Three models were adopted: Model 1, without any adjustments; Model 2, adjusted for age, gender, and energy intake; and Model 3, adjusted similar to Model 2 plus protein intake, fat intake, carbohydrate intake, physical activity (MET, hours/week), smoking status (non-smoker, ex-smoker, and current smoker), alcohol drinking (yes or no), income (tertile), urbanization index (tertile), education (low, medium, and high), and BMI (<18.5, 18.5–23.9, 24.0–27.9, or ≥28 kg/m^2^). The sample size in the fully adjusted model was 2194. The multiplicative interaction between the selenium intake and the potential influencing factors (age, sex, region, alcohol drinking, smoking, hypertension, diabetes, hyperlipidemia, BMI, and physical activity) was analyzed by adding the product of the variables in the regression model. We also performed a subgroup analysis of these factors. A *p* < 0.05 was considered to be statistically significant for all statistical analyses.

## 3. Results

Table 1 describes the sample characteristics of selenium intake by quartiles. A total of 5381 participants were included in the study and 942 (17.01%) participants had CKD. A univariate analysis showed that the energy intake, carbohydrate intake, fat intake, and protein intake were positively correlated with the selenium intake whereas age was inversely correlated with the selenium intake. Significant differences in the selenium intake by sex, alcohol consumption, smoking, income, urbanization, region, BMI, and education level were found. The prevalence of diabetes and hyperlipidemia was not statistically associated with the selenium intake, but both hypertension and CKD were found to be at low rates in the high selenium group.

Across the selenium intake quartiles, the prevalence of CKD was 23.33%, 20.32%, 14.98%, and 9.25% (Table 2), respectively. In a logistic regression analysis of CKD and dietary selenium, all three models showed a negative association between the selenium intake and the prevalence of CKD in the highest selenium intake group. In the fully adjusted model, Model 3, the ORs for prevalent CKD were 1.00, 1.09 (95% CI 0.69–1.73), 0.82 (95% CI 0.49–1.38), and 0.43 (95% CI 0.22–0.85) across the quartiles of selenium intake, respectively. The trend was statistically significant. We attempted to include dietary selenium density (µg/1000 kcal) into the logistic regression analysis; the results showed an even stronger association between selenium and CKD (Supplementary Table S1).

No interactions were observed between the selenium intake and age, sex, region, alcohol drinking, hypertension, diabetes, hyperlipidemia, BMI, and physical activity level for the CKD odds (Table 3). Amongst the people aged 60 years or older, the prevalence of CKD was 0.23 (95% CI 0.08–0.67) in the group with the highest selenium intake compared with that in the lowest. Similarly, in the subgroups of females (OR: 0.36, 95% CI 0.14–0.95), non-/ex-smokers (OR: 0.36, 95% CI 0.16–0.81), non-alcohol drinking (OR: 0.32, 95% CI 0.14–0.73), non-hypertensive (OR: 0.38, 95% CI 0.15–0.99), non-diabetic (OR: 0.43, 95% CI 0.22–0.86), non-hyperlipidemia (OR: 0.44, 95% CI 0.19–1.02), and high levels of physical activity (OR: 0.12, 95% CI 0.02–0.61), the highest selenium group was independently associated with a low odds of CKD. A significant negative relationship between the selenium intake and CKD was found in the overweight (OR: 0.09, 95% CI 0.02–0.46), but not obese, individuals. Notably, a significant north–south difference was observed in the relationship between selenium intake and CKD. The selenium intake in the second quartile group was potentially a statistically significant odds factor for CKD in the north (OR: 3.80, 95% CI 1.03–13.98).

## 4. Discussion

The study subjects included 5381 middle-aged and elderly people from the CHNS project in 2009. Currently, the international recommended intake of dietary selenium for adults varies from 26 µg/day to 75 µg/day [35,36]. In our study, the dietary selenium intake was generally lower than the recommended intake (60 µg/day) set by China [37]. The prevalence of CKD in people aged over 45 years (17.01%) was slightly higher than that in US adults (13.4%) for the same period [18]. Given that our study subjects were of a different race and the average age was older, this difference was considered to be acceptable.

In the fully adjusted model, Model 3, a high dietary selenium intake may have been a protective factor for CKD; this result supported previous research [22]. According to research in Taiwan, plasma selenium was positively correlated with the eGFR and the odds of CKD were 3.35 times higher in the low selenium group than in the high selenium group [3]. In a randomized double-blind placebo-controlled prospective trial of 215 older adults, a significantly better renal function was found in the treatment groups provided with supplements of selenium and coenzyme Q10 than in the control group [25]. Selenium Supplementation can protect the kidneys from renal ischemia [38], heavy metals [3], and mycotoxins [39]. No association between selenium and renal function has been reported. A population-based cross-sectional study in China that included 3553 subjects reported no correlation between serum/urinary selenium and renal function [40]. Fang et al. [5] used the evidence strength criteria proposed by the WHO to classify all the articles on the health effects of selenium included in Medline, Google Scholar, and the China National Knowledge Network (CNKI). No health effects of selenium on CKD were found among the eight beneficial and one adverse health effects identified.

Selenium is not an antioxidant per se, but it acts as a cofactor of antioxidant enzymes [9]. The mechanism of selenium affecting CKD may be related to the following aspects. First, selenium increases the activity of GPx3 in the body [9], which may protect the kidney function by preventing mitochondrial damage caused by elevated oxidative stress [23]. Second, selenium has a positive effect on phagocytes, T cells, and immunoglobulin, thereby enhancing human immunity [8]. Third, selenium affects renal hemodynamics by playing an important role in the thyroid function [41]. Dyslipidemia caused by hypothyroidism may adversely affect the recovery of CKD [42]. In addition, the Wnt/β-catenin pathway may play an important role in renal fibrosis caused by a selenium deficiency [43].

The effect of selenium on CKD varies in different populations. The protective effect of high selenium on CKD was more significant in people aged 60 years and older than among their younger counterparts. This result suggests the significance of selenium Supplementation for middle-aged and elderly people susceptible to CKD. More complex antioxidant mechanisms in women may contribute to a greater sensitivity to selenium [44]. These factors may contribute to the gender difference observed in longevity, given that women live longer than men. Unhealthy lifestyles such as alcohol consumption, smoking, and sedentary behavior [18] were not only direct risk factors for CKD, but also interfered with the normal physiological function of selenium in protecting the kidneys. We found that the odds of CKD decreased with an increased selenium intake in overweight individuals. A controlled clinical trial showed that selenium supplementation reinforced the effects of diet on overweight/obese individuals and increased lean muscle mass [45]. This mechanism may also have a positive influence on the protective effect of selenium on CKD. This may not be the case in obese people because they suffer from high levels of oxidative stress [46] often accompanied by multiple metabolic diseases (including obesity itself [47]), which are strongly correlated with the selenium levels in the human body [48,49]. The results found in our study in our disease subgroups supported this view. Interestingly, regional differences were found in the association between selenium intake and CKD. Consistent with another study on selenium and hypertension, this difference may be due to the uneven distribution of selenium in the soil [13].

Grains, meat, and dairy products contribute to most of the dietary selenium intake [50]. Protein-rich foods contain high levels of selenium and they are associated with the high bioavailability of selenium [50].

In addition to the CKD-EPI equation, the Modification Of Diet In Renal Disease (MDRD) study equation [51] is commonly used to estimate the eGFR. The MDRD equation yielded lower estimates of the eGFR than the CKD-EPI equation [52]. The mean difference between the two equations was small when the eGFR levels were low, but large when the renal function was slightly reduced or near normal [52]. Studies have shown that the CKD-EPI equation is more accurate than the MDRD equation [53], especially in classifying individuals at risk of CKD in middle-aged people with a normal or near normal renal function [54]. We used standardized serum creatinine values in the MDRD equation for estimating the eGFR [51] and the results were similar to the CKD-EPI equation (Supplementary Table S2). The two methods yielded consistent results, confirming the possible benefit of selenium on the onset of CKD.

## 5. Conclusions

In conclusion, an adequate selenium intake may have a positive effect on the prevention and treatment of CKD. This role is better served by adhering to good lifestyle habits (e.g., continued physical activity, not drinking alcohol, and not smoking) and the absence of other metabolic diseases. The negative association between selenium and CKD found only in overweight people deserves a further investigation. It is also prudent to be cautious about regional differences when providing selenium supplements.

In this study, which was based on a large population sample, we have addressed the gap in domestic research on dietary selenium and CKD and innovatively explored a new method for predicting selenium intake. This study had the following shortcomings. First, we only included data from 2009. The cross-sectional study design did not allow us to specify the causality of all the observed associations, so further evidence is needed. Second, the determination of selenium in human studies is complex and the determined selenium levels can vary significantly even for healthy individuals [55]. Selenoprotein may be included as an indicator in the future [56]. Third, our study lacked data on albuminuria, which is an earlier marker of kidney failure.

## Figures and Tables

**Table 1:** Sample characteristics by quartiles of Se intake: CHNS (*n* = 5381).

Factor	Q1	Q2	Q3	Q4	*p*-Value
N	1350	1368	1322	1341	
Se intake (µg/day), mean (SD)	21.5 (4.8)	33.1 (2.8)	43.8 (3.7)	67.0 (14.0)	
Se intake (µg/day), range	7.8~28.2	28.4~37.8	38~50.8	51~114.2	
Age, mean (SD)	61.8 (10.8)	60.1 (10.1)	58.3 (9.3)	57.0 (9.0)	**<0.01**
Energy intake (kcal/day), mean (SD)	1656.2 (463.8) (*n =* 1337)	2009.9 (512.7) (*n =* 1367)	2215.3 (566.9) (*n =* 1320)	2529.4 (634.1) (*n =* 1327)	**<0.01**
Carbohydrate intake (g/day), mean (SD)	238.4 (80.3) (*n =* 1337)	277.3 (87.0) (*n =* 1367)	299.2 (93.8) (*n =* 1321)	340.6 (108.8) (*n =* 1337)	**<0.01**
Fat intake (g/day), mean (SD)	56.7 (27.4) (*n =* 1341)	70.9 (29.7) (*n =* 1359)	77.9 (32.9) (*n =* 1303)	86.6 (34.6) (*n =* 1313)	**<0.01**
Protein intake (g/day), mean (SD)	44.9 (13.1) (*n =* 1339)	58.2 (13.5) (*n =* 1368)	69.3 (16.5) (*n =* 1321)	83.4 (20.1) (*n =* 1313)	**<0.01**
Physical activity (MET h/week), mean (SD)	172.1 (111.2) (*n =* 514)	169.5 (105.1) (*n =* 553)	165.7 (107.3) (*n =* 589)	177.4 (100.2) (*n =* 661)	0.25
Sex					**<0.01**
Male	484 (35.9%)	616 (45.0%)	645 (48.8%)	785 (58.5%)	
Female	866 (64.1%)	752 (55.0%)	677 (51.2%)	556 (41.5%)	
Alcohol					**<0.01**
No	1046 (77.7%)	959 (70.1%)	884 (66.9%)	790 (58.9%)	
Yes	301 (22.3%)	409 (29.9%)	438 (33.1%)	551 (41.1%)	
Smoker					**<0.01**
Non-smoker	997 (74.0%)	935 (68.3%)	889 (67.3%)	814 (60.7%)	
Ex-smoker	57 (4.2%)	51 (3.7%)	49 (3.7%)	74 (5.5%)	
Current smoker	293 (21.8%)	382 (27.9%)	382 (28.9%)	453 (33.8%)	
Income					**<0.01**
Low	554 (42.7%)	437 (33.2%)	400 (30.8%)	347 (26.3%)	
Medium	439 (33.9%)	449 (34.1%)	429 (33.1%)	434 (33.0%)	
High	303 (23.4%)	430 (32.7%)	468 (36.1%)	536 (40.7%)	
Urbanization					**<0.01**
Low	553 (41.0%)	475 (34.7%)	377 (28.5%)	402 (30.0%)	
Medium	432 (32.0%)	477 (34.9%)	438 (33.1%)	453 (33.8%)	
High	365 (27.0%)	416 (30.4%)	507 (38.4%)	486 (36.2%)	
Region					**<0.01**
North	469 (34.7%)	523 (38.2%)	556 (42.15)	741 (55.3%)	
South	881 (65.3%)	845 (61.8%)	766 (57.9%)	600 (44.7%)	
Education					**<0.01**
Low	283 (21.1%)	299 (21.9%)	264 (20.0%)	266 (19.9%)	
Medium	236 (17.6%)	351 (25.7%)	360 (27.3%)	424 (31.6%)	
High	198 (14.8%)	254 (18.6%)	299 (22.7%)	325 (24.3%)	
Unknown	625 (46.6%)	461 (33.8%)	397 (30.1%)	325 (24.3%)	
BMI					**<0.01**
Lower	123 (9.3%)	85 (6.3%)	61 (4.7%)	32 (2.4%)	
Normal	699 (53.0%)	705 (52.6%)	644 (49.4%)	635 (48.1%)	
Overweight	380 (28.8%)	423 (31.6%)	450 (34.5%)	485 (36.7%)	
Obesity	118 (8.9%)	127 (9.5%)	149 (11.4%)	168 (12.7%)	
CKD					**<0.01**
No	1035 (76.7%)	1090 (79.7%)	1124 (85.0%)	1217 (90.8%)	
Yes	315 (23.3%)	278 (20.3%)	198 (15.0%)	124 (9.2%)	
Hypertension					**<0.01**
No	694 (51.4%)	662 (48.4%)	671 (50.8%)	750 (55.9%)	
Yes	656 (48.6%)	706 (51.6%)	651 (49.2%)	591 (44.1%)	
Diabetes					0.13
No	1227 (90.9%)	1206 (88.2%)	1184 (89.6%)	1205 (89.9%)	
Yes	123 (9.1%)	162 (11.8%)	138 (10.4%)	136 (10.1%)	
Hyperlipidemia					0.10
No	870 (64.4%)	822 (60.1%)	807 (61.1%)	843 (62.9%)	
Yes	480 (35.6%)	545 (39.9%)	514 (38.9%)	498 (37.1%)	

The statistical data are shown as means (SD) for continuous variables and counts (percentages) for categorical variables. The *p*-values were calculated from an ANOVA or a chi-squared test. CKD, chronic kidney disease; BMI, body mass index. Significant associations are shown in bold type (*p* < 0.05). We defined Heilongjiang, Liaoning, Shandong, and Henan as the northern regions and Jiangsu, Hubei, Hunan, Guizhou, and Guangxi as the southern regions.

**Table 2:** Logistic regression analysis of CKD status and dietary selenium (quartile) in adults.

	Q1	Q2	Q3	Q4	*p* for Trend
Se intake (µg/day), mean (SD)	21.5 (4.82)	33.1 (2.79)	43.8 (3.70)	67.0 (13.97)	
case	1350	1368	1322	1341	
Prevalence	23.33%	20.32%	14.98%	9.25%	
Model 1	1	0.838 (0.698–1.005)	**0.579 (0.475–0.705)**	**0.335 (0.268–0.419)**	**<0.001**
Model 2	1	1.057 (0.852–1.311)	0.897 (0.708–1.137)	**0.575 (0.435–0.759)**	**<0.001**
Model 3	1	1.092 (0.69–1.729)	0.818 (0.485–1.378)	**0.427 (0.216–0.845)**	**0.017**

Model 1, without any adjustments; Model 2, adjusted for age, gender, and energy intake; Model 3, adjusted as for Model 2 plus protein intake, fat intake, carbohydrate intake, physical activity (MET, hours/week), smoking status (non-smoker, ex-smoker, current smoker), alcohol drinking (yes or no), income (tertile), urbanization index (tertile), education (low, medium, high), and BMI (< 18.5, 18.5–23.9, 24.0–27.9, or ≥ 28 kg/m^2^). Bold: statistically significant.

**Table 3:** Subgroup analyses of the association between selenium intake (quartile) and prevalent CKD.

	Q1	Q2	Q3	Q4	*P* for Interaction
Age					0.327
< 60	1.00	1.01 (0.53–2.01)	0.86 (0.42–1.77)	0.58 (0.24–1.43)	
≥ 60	1.00	1.00 (0.53–1.90)	0.55 (0.25–1.19)	**0.23 (0.08–0.67)**	
Sex					0.582
Male	1.00	0.92 (0.47–1.81)	0.82 (0.38–1.73)	0.41 (0.16–1.04)	
Female	1.00	0.99 (0.53–1.82)	0.54 (0.26–1.12)	**0.36 (0.14–0.95)**	
Region					0.388
North	1.00	**3.80 (1.03–13.98)**	1.35 (0.30–6.11)	2.36 (0.49–11.43)	
South	1.00	0.97 (0.58–1.62)	0.94 (0.51–1.73)	0.56 (0.24–1.31)	
Alcohol Drinking					0.826
No	1.00	0.90 (0.53–1.53)	0.59 (0.32–1.09)	**0.32 (0.14–0.73)**	
Yes	1.00	1.12 (0.46–2.75)	0.74 (0.27–2.08)	0.53 (0.16–1.76)	
Smoker					0.239
Non-/ex-smoker	1.00	0.89 (0.52–1.55)	**0.52 (0.27–0.99)**	**0.36 (0.16–0.81)**	
Current smoker	1.00	1.22 (0.54–2.79)	1.04 (0.41–2.66)	0.38 (0.11–1.30)	
Hypertension					0.756
No	1.00	0.87 (0.46–1.64)	0.57 (0.28–1.19)	**0.38 (0.15–0.99)**	
Yes	1.00	1.14 (0.58–2.24)	0.79 (0.36–1.71)	0.42 (0.16–1.14)	
Diabetes					0.248
No	1.00	0.86 (0.53–1.38)	0.66 (0.38–1.14)	**0.43 (0.22–0.86)**	
Yes	1.00	5.09 (0.65–41.13)	1.11 (0.11–11.21)	0.13 (0.004–4.32)	
Hyperlipidemia					0.552
No	1.00	0.94 (0.54–1.66)	**0.52 (0.27–1.01)**	**0.44 (0.19–1.02)**	
Yes	1.00	1.42 (0.63–3.20)	1.20 (0.49–2.92)	0.34 (0.10–1.20)	
BMI					0.720
Lower	1.00	0.88 (0.16–4.91)	1.66 (0.25–10.96)	2.67 (0.22–32.32)	
Normal	1.00	1.17 (0.65–2.13)	0.83 (0.42–1.63)	0.43 (0.17–1.07)	
Overweight	1.00	0.74 (0.30–1.84)	**0.24 (0.07–0.78)**	**0.09 (0.02–0.46)**	
Obesity	1.00	0.32 (0.025–4.16)	0.89 (0.12–6.84)	0.79 (0.10–6.03)	
Physical activity					0.674
Low	1.00	1.35 (0.68–2.67)	0.93 (0.40–2.13)	0.54 (0.18–1.63)	
Medium	1.00	1.09 (0.48–2.48)	0.70 (0.29–1.70)	0.48 (0.16–1.41)	
High	1.00	0.57 (0.21–1.54)	0.32 (0.10–1.06)	**0.12 (0.02–0.61)**	

Model adjusted for age, gender, energy intake, protein intake, fat intake, carbohydrate intake, physical activity, smoking status, alcohol drinking, income, urbanization index, education, and BMI. Stratification variables were not adjusted in the corresponding models. Bold: statistically significant.

## Data Availability

The datasets generated during and/or analyzed during the current study are available in the CHNS repository, https://www.cpc.unc.edu/projects/china (accessed on 24 April 2022).

## Supplementary Materials

The following supporting information can be downloaded at: https://www.mdpi.com/article/10.3390/nu14132695/s1, Table S1: Logistic regression analysis of CKD status and dietary selenium density (quartile) in adults; Table S2: Logistic regression analysis of CKD status (measured by MDRD equation) and dietary selenium (quartile) in adults (≥45 years old).
