# Supplementary material for: Association between Selenium Status and Chronic Kidney Disease in Middle-Aged and Older Chinese Based on CHNS Data
Source: Nutrients. 2022 Jun 28;14(13):2695. doi: 10.3390/nu14132695 (PMC9269073; doi:10.3390/nu14132695)
Supplement: Supplementary file 1 [file nutrients-14-02695-s001.zip › Supplementary Table S1.pdf]

**Supplementary Table S1.** Logistic regression analysis of CKD status and dietary selenium density (quartile) in adults.

| | Q1 | Q2 | Q3 | Q4 | P for Trend |
|---|---|---|---|---|---|
| Se intake (µg/1000kcal), mean (SD) | 11.8(2.0) | 16.4(1.1) | 20.9(1.5) | 30.3(6.2) | |
| case | 1338 | 1338 | 1338 | 1337 | |
| prevalence rate | 19.22% | 18.85% | 16.02% | 13.95% | |
| Model 1 | 1 | 0.98(0.80-1.19) | **0.80(0.66-0.98)** | **0.68(0.55-0.84)** | **<0.001** |
| Model 2 | 1 | 1.01(0.81-1.27) | 0.86(0.69-1.08) | **0.65(0.51-0.82)** | **<0.001** |
| Model 3 | 1 | **0.62(0.40-0.97)** | **0.61(0.38-0.99)** | **0.36(0.19-0.66)** | **0.001** |

Model 1 adjusted for none; Model 2 adjusted for age, gender and energy intake; Model 3 adjusted as for model 1 plus protein intake, fat intake, carbohydrate intake, physical activity (MET, hours/week), smoking status (non-smoker, ex-smokers, current smokers), drinking (yes or no), income (tertile), urbanization Index (tertile), education (low, medium, high), and BMI (<18.5, 18.5–23.9, 24.0–27.9, or>=28 kg/m2 ). Bold: statistically significant.
