# Supplementary material for: Association between Selenium Status and Chronic Kidney Disease in Middle-Aged and Older Chinese Based on CHNS Data
Source: Nutrients. 2022 Jun 28;14(13):2695. doi: 10.3390/nu14132695 (PMC9269073; doi:10.3390/nu14132695)
Supplement: Supplementary file 1 [file nutrients-14-02695-s001.zip › Supplementary Table S2.pdf]

Supplementary Table S2. Logistic regression analysis of CKD status and dietary selenium (quartile) in adults (>=45 years old).

	**Q1**	**Q2**	**Q3**	**Q4**	**P for Trend**
Se intake (μg/day), mean (SD)	21.5(4.82)	33.1(2.79)	43.8(3.70)	67.0(13.97)	
case	1350	1368	1322	1341	
Prevalence	22.45%	20.28%	15.72%	9.83%	
Model 1	1	0.88(0.73-1.05)	**0.67(0.55-0.81)**	**0.37(0.30-0.46)**	**<0.001**
Model 2	1	1.09(0.89-1.34)	1.00(0.80-1.25)	**0.63(0.49-0.82)**	**0.002**
Model 3	1	1.05(0.68-1.61)	0.97(0.61-1.55)	**0.43(0.23-0.80)**	**0.021**

Model 1 adjusted for none; Model 2 adjusted for age, gender and energy intake; Model 3 adjusted as for model 1 plus protein intake, fat intake, carbohydrate intake, physical activity (MET, hours/week), smoking status (non-smoker, ex-smokers, current smokers), drinking (yes or no), income (tertile), urbanization Index (tertile), education (low, medium, high), and BMI (<18.5, 18.5–23.9, 24.0–27.9, or>=28 kg/m2).Bold: statistically significant.
